# Highly Active Cranberry’s Polyphenolic Fraction: New Advances in Processing and Clinical Applications

**DOI:** 10.3390/nu13082546

**Published:** 2021-07-26

**Authors:** Alessandro Colletti, Luciano Sangiorgio, Alma Martelli, Lara Testai, Arrigo F. G. Cicero, Giancarlo Cravotto

**Affiliations:** 1 Department of Drug Science and Technology, University of Turin, 10125 Turin, Italy; alessandro.colletti@unito.it; 2 Department of Pediatric Urology, SS. Antonio and Biagio and Cesare Arrigo Hospital, 15121 Alessandria, Italy; Lsangiorgio@ospedale.al.it; 3 Department of Pharmacy, University of Pisa, Via Bonanno 6, 56120 Pisa, Italy; alma.martelli@unipi.it (A.M.); lara.testai@unipi.it (L.T.); 4 Interdepartmental Research Centre “Nutraceuticals and Food for Health (NUTRAFOOD)”, University of Pisa, 56120 Pisa, Italy; 5 Interdepartmental Research Centre of Ageing, Biology and Pathology, University of Pisa, 56120 Pisa, Italy; 6 Medical and Surgical Sciences Department, University of Bologna, 40138 Bologna, Italy; arrigo.cicero@unibo.it; 7 IRCCS AOU S. Orsola-Malpighi, 40138 Bologna, Italy; 8 World-Class Research Center “Digital Biodesign and Personalized Healthcare”, Sechenov First Moscow State Medical University, 119048 Moscow, Russia

**Keywords:** cranberry, *Vaccinum macrocarpon*, urinary tract infections, nutraceutical, extraction techniques, proanthocyanidins

## Abstract

Cranberry is a fruit originally from New England and currently growing throughout the east and northeast parts of the USA and Canada. The supplementation of cranberry extracts as nutraceuticals showed to contribute to the prevention of urinary tract infections, and most likely it may help to prevent cardiovascular and gastroenteric diseases, as highlighted by several clinical trials. However, aiming to validate the efficacy and safety of clinical applications as long-term randomized clinical trials (RCTs), further investigations of the mechanisms of action are required. In addition, a real challenge for next years is the standardization of cranberry’s polyphenolic fractions. In this context, the optimization of the extraction process and downstream processing represent a key point for a reliable active principle for the formulation of a food supplement. For this reason, new non-conventional extraction methods have been developed to improve the quality of the extracts and reduce the overall costs. The aim of this survey is to describe both technologies and processes for highly active cranberry extracts as well as the effects observed in clinical studies and the respective tolerability notes.

## 1. Introduction

*Vaccinum macrocarpon* (Ait. Ericaceae) called “large cranberry”, “North American cranberry” “bearberry”, or simply “cranberry” is a fruit originally from New England and currently grows throughout the east and northeast parts of the USA and Canada. The USA produces 436.691 tons/year of cranberries equal to 58% of the world’s total cranberry production, with $3.5 billion estimated in economic activity [1,2,3]. However, only 3–5% of cranberries are used for fresh fruit consumption, while 95–97% are destinated for processing, which includes both the food and nutraceutical industries [2]. In fact, cranberries are known as a rich source of phenolic compounds and used for a long time in traditional folk medicine, especially for the treatment of bladder and kidney ailments [4]. Starting from the second half of the 20th century the use of *V. macrocarpon* juice to treat urinary tract infections (UTIs) and wounds has been reported in different manuals of phytotherapy. According to subsequently observational and interventional studies in humans, consumption of cranberry demonstrated to be associated with beneficial effects firstly in UTIs prevention, but also in other conditions [5]. In this regard, cranberries possess antioxidant and anti-inflammatory activity, and its supplementation have shown to promote overall gut and oral health and reduce or prevent some cardiovascular risk factor or chronic conditions (e.g., hyperglycaemia, dyslipidaemia, diabetes type II, cardiovascular diseases) and even cancer [6,7,8]. During the last years, the new trend of studies has focused the attention also on the effects of cranberry on gut microbiota composition and gastrointestinal health [9]. In this sense, the consumption of 2 bottles of cranberry juice (each of 250 mL) for 90 days was found to suppress *Helicobacter pylori* infection of susceptible people [10].

After a description of the main bioactive components of cranberry, this review will describe the state of the art of the main extraction and purification methods of cranberry, discussing the advantages and limitations of these strategies. Clinical applications that have arisen from RCTs are also discussed. Finally, future perspectives for cranberry extracts will be presented.

## 2. Methods

The literature search has been performed using the most relevant databases recognized for medical scientific literature, including PubMed, MEDLINE (National Library of Medicine, Bethesda, Maryland, MD, USA; January 1970 to June 2021), the Cochrane Register of Controlled Trials (The Cochrane Collaboration, Oxford, UK) with access to Scopus, EMBASE, and ClinicalTrials.gov. The terms used for the electronic search strategy were ‘cranberry’, ‘proanthocyanidins’, ‘urinary tract infections’, ‘*Vaccinum macrocarpon*’, ‘extraction techniques’, and ‘clinical trial’. The eligible papers must be published in English. Randomized clinical trials have been preferred and included wherever possible, although in some minor conditions both open-label and animal studies were considered due to the lack of controlled studies. The work is characterized by a first general introduction and a description of the phytochemical composition of cranberry, followed by a description of the technologies and processes for highly active cranberry extracts, the effects observed in clinical studies, and the respective tolerability notes. The Declaration of Interest forms related to real or potential sources of conflicts of interest were compiled by the drafting and review groups.

### 2.1. Phytochemical Composition of Cranberry

*V. macrocarpon* phytochemical composition is complex and includes many polyphenols like A-type procyanidins (PACs), anthocyanins, benzoic acid, but also terpenes like ursolic acid (UA). The amounts of different classes of phytochemicals in cranberry fresh fruit, dried fruit, juice, and sauce are synthesized in Table 1. PACs represent about 85% of the total weight of the flavan-3-ols [11] and comprise a group of different chemical structures based on the common constitutive unit represented by the (−)-epicatechin. The several types of PACs derived by different types of linkage and degree of polymerization, in particular the presence of A-type or B-type, influences the efficacy of the extract against urinary tract infections (UTI). Indeed, the A-type is significantly more effective than the B-type in inhibiting *Escherichia coli* adhesion to uroepithelial cells [12]. Moreover, while PACs are contained in high amounts in several plant foods such as apples or grapes, A-type PACs are contained in high amounts only in cranberries and lingonberry (*Vaccinum vitis-idaea* L.) [11]. The anthocyanins mainly present in cranberries are cyanidin and peonidin glycosides. Among them, four anthocyanins, i.e., cyanidin-3-galactoside, cyanidin-3-arabinoside, peonidin-3-galactoside, and peonidin-3-arabinoside have been identified as the most representative while cyanidin-3-glucoside, peonidin-3-glucoside, and other various anthocyanins are present only in small amounts [13]. Anthocyanins are mainly responsible for the colour of the berries and the naturally occurring glycosylation in position 3, increasing the stability of its aglycone portion, the anthocyanidins, which is highly unstable [13].

Phenolic acids are not specific components; indeed, it is present in many other plant food and berries. In cranberries, phenolic acids are mainly represented by hydroxybenzoic and hydroxycinnamic acids while ellagic acid and ellagitannins have not been detected. The hydroxybenzoic acids are the most abundant and benzoic acid is more prevalent than *p*-hydroxybenzoic, *o*-hydroxybenzoic, and 2,4-dihydroxybenzoic acids. As concerns the hydroxycinnamic acids, cranberries contain *p*-coumaric, caffeic, ferulic, and sinapic acids [20].

Cranberry fruits also contain phytochemicals not included in the polyphenols class such as triterpenoids compounds like ursolic, oleanolic, and betulinic acids. Among them, the most abundant is the ursolic acid which is present also in the peels of several fruits and which has anti-inflammatory recognized properties [21] but cranberry fruits also contain two rare derivatives of UA such as *cis-* and *trans*-3-*O*-*p*-hydroxycinnamoyl ursolic acid. On the other hand, in a study focused on cranberry juice fractions tested through a bacterial adherence assay, two coumaroyl iridoid glycosides and a depside have been identified [22].

Among berries and plant food, *V. macrocarpon* is probably the richest species for flavonols and the most abundant flavonols, in cranberries are represented by the glycosylated form of quercetin, myricetin, and a small amount of kaempferol. In particular, quercetin 3-galactoside represents the predominant flavanol, even if other glycosides are present in traces [17].

The exact amount of each bioactive compound in cranberry depends on the variety of American cranberry which is analysed and from the exact phase of the plant life. In general, PACs and flavonols have been found high in the earliest stage and decrease during the growth of the fruits. The decrease of PACs is more rapid than the decline of flavanols. Anthocyanins production starts when cranberry fruit has finished its growth and continues during ripening [14].

All the phytochemicals are present in the cranberry fresh fruits but often cranberries are consumed in processed forms to obtain juice, sauce or dried fruits, and these processes could affect the content of bioactive compounds (Table 1). Anthocyanins are particularly affected, and their loss could reach 50% of the total amount due to the removal of skin, and seeds, high temperatures and oxidation of polyphenols. PACs and flavonols are more resistant against the high temperatures and during pasteurization but not against the very high heat used during the process necessary to obtain cranberry powders [23].

### 2.2. Extraction Methods

Several types of cranberry products have been tested in pre-clinical and clinical trials even if data regarding the comparison of the relative composition of the extracts and the bioactivity for different products is still few or lacking [7].

**Figure 1:**
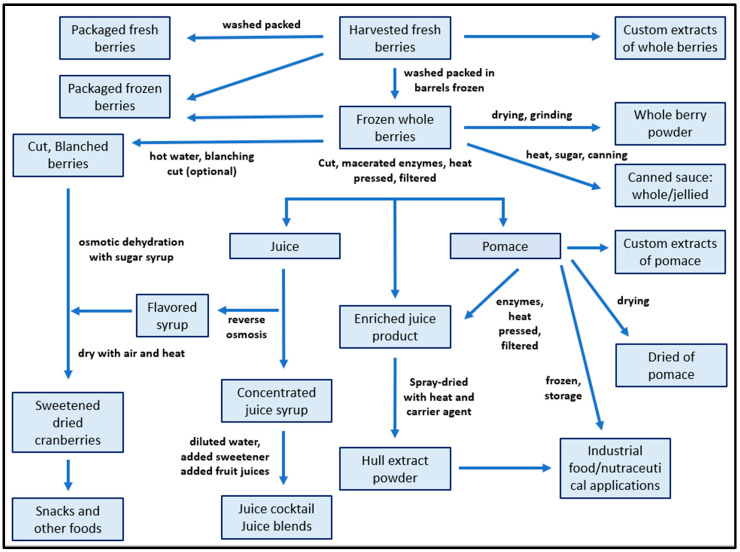
General processing steps and relative relationship for different cranberries materials (Adapted from *Coleman* et al. [7]).

American cranberry is rarely consumed as fresh fruit, due to the sour, astringent flavour of the berry [14]. For this reason, a wide range of cranberry products are available on the market (Figure 1) for both the consumers and the researchers with different chemical profiles, according to the extraction methods used.

The most common use of cranberry is the production of juice, which causes an increase in food industry waste, well known as pomace (berry press residues) that contain berry skin and seeds [24]. In a concept of circular economy, the use of cranberry by-products represents an interesting strategy for the valorisation of food industry wastes, recovering from the berry press residues different nutraceutical molecules, such as the phenolic compounds (Table 2) and the oligosaccharides fractions (usually discarded), which are known to have health properties [24]. However, the optimisation of the extraction technique is necessary in order to selectively extract the molecules of interest, with an acceptable yield. In fact, the extraction of phenolic compounds from cranberry seeds is often difficult, especially using “green solvents” and unconventional extraction methods [25]. Pomace may be used also as an animal feed additive [26].

Depending on the nature of the chemical and physical extraction processes and the sequence of processing steps adopted, it is possible to obtain several cranberry extracts with different bioactive components (Table 3). Whole berries are generally stored frozen after harvest and a rapid wash, increasing the storage period and allowing subsequent treatments for several months after the fruit has been harvested [28].

Cranberry juice is extracted by pressing or decanting from frozen berries which have been cut and macerated and include the separation of solid and liquid fruit components, followed by centrifugation and filtering, in order to remove the suspended solid particulates [29]. The freezing, cutting, and maceration processes are important to increase the extraction of phenolic components, improving the surface area of fruits by breaking down cell wall components [30].

After the separation of solid and liquid fruit components, the juice is particularly rich in water-soluble molecules including several flavonoids and organic acids (tartaric, fumaric, citric, and shikimic acid) as well as sugars (fructose, glucose, sucrose), while the pomace is composed mainly of cranberry skins and seeds is rich in proteins, insoluble polymers, and polyphenols adhered within the physical structures of such residues [7]. The extraction of bioactive components can be improved also using a mixture of pectinase enzymes in addition to water and heat to biochemically degrade cell wall structures [31,32].

In general, the liquid achieved from the juice production undergoes the process of pasteurization with the use of heat to facilitate the long-term storage. The concentration of juice exploits the mechanism of reverse osmosis or counter current chromatography, obtaining the cranberry juice concentrate also known as cranberry syrup with a remarkably high concentration of solute (50 Brix) [33]. Concentrated juice syrup is then diluted with water and sweetened or blended with other fruit juices to obtain respectively sweet cranberry juice and mixed fruit juices [29].

Cranberry juice can be treated with proprietary methods to remove sugars, especially for nutraceuticals preparations, in order to reduce the caloric content of juice and avoid any problems with gluco-intolerant patients [34,35].

Fresh berries can also be processed to obtain dried cranberries, canned cranberry sauce, and other foods with added cranberry materials. Sweetened dried cranberries prepared from the frozen, cut berries are instead used in food industries [28].

However, during the drying process, there is a partial loss of nutritional value as a consequence of exposure of cranberry fruits to high temperatures and air for a long time. In this regard, during the extrusion process, characterized by the exposition of cranberries at high-temperature for a short-time, in order to reduce the moisture and then prolong the shelf-life, a significant loss of total anthocyanins have been observed [24].

For this reason, and in addition to reducing energy expenditures, new unconventional technologies and pre-treatment have been developed [36]. The study by Nowacka et al. analysed the quality of microwave-vacuum dried, osmodehydrated cranberries processed by the means of blanching and ultrasound or blanching followed by pulsed electric field and sonication in comparison with the traditionally treated material. the microwave-vacuum drying process was demonstrated to be much shorter (25–38 min) than convective drying (over 13 h). in addition, the content of bioactive molecules such as polyphenols including anthocyanins and flavonoids was similar or higher under ultrasound and pulsed electric field. analogously with osmodehydration and drying with a pre-sonication if compared with osmodehydrated cranberry fruits subjected to convective drying (reference samples) [37].

An interesting technique to reduce deteriorating reactions and increase the shelf life is represented by the food coating, which is characterized by the application of edible layers on the surface of cranberries. In this regard, Lozano-Navarro et al. [38] reported the antimicrobial impact when 0.5% of cranberry extract was added to chitosan and starch-based film.

An interesting product from cranberry pomace or enriched juice (from the whole berries) is the hull extract powder, which can be used both for food and nutraceutical applications. Starting from the pomace or the whole berries cut, after pressing and filtering, the enriched juice is treated with additional enzymes and heat beyond those typically used for juice production. After that, the resulting enriched juice extract is typically spray-dried to obtain the hull extract powder [7].

The composition of cranberry extracts is extremely variable, depending on several factors, such as the treatments with different enzymes, temperature and time conditions, and the concentration and drying steps that affect the qualitative-quantitative composition of the final extracts [39]. Considerable evidence suggests that flavonoids could be easily degraded during processing due to different factors which affect their stability (temperature, light, pH, and the presence of endogenous enzymes, such as polyphenol oxidase or glycosidases). Flavonoids are generally stable at a lower pH, reducing the conversion from the stable flavilium cation to less stable carbinol pseudobases or quinodal bases which are more easily oxidized. Even the presence of polyphenol oxidase and/or glycosidase can reduce the stability of anthocyanins reacting with simple phenolics to form quinones or cleaving the sugar from many flavonoids respectively. Several studies showed that flavonoid stability appears to be dependent primarily on the sugar attached rather than the aglycone, and glucosides are more stable than galactosides, which are in turn more stable than arabinosides [40,41]. Despite the little information available on the relationship between the glycosylation and the enteric bioaccessibility of cranberry polyphenols, it seems that this phenomenon may improve the bioavailability of several phenolic compounds probably by an enhanced mechanism of absorption that involve the use of the sodium dependent glucose cotransporter SGLT1 [42].

However, the great variability of bioactive molecules present in the extracts could influence the results of cranberry effects administered in clinical practice. In addition, even if a major part of the clinical studies has investigated the effects of cranberry as a nutraceutical, marketed in the form of dry extracts (some of these titrated and standardized in phenolic compounds) in different dosage forms (syrups, sachets, capsules, tablets, etc.), the lack of information which describe the methods used to obtain the extracts make impossible to predict the difference of bioactive compounds which may be present in the final product [43,44].

### 2.3. Unconventional Extraction Techniques

A consolidated method to extract polyphenols from cranberry pomace provides the use of petrochemical-based solvents, including methanol, acetone, ethyl acetate, and chloroform. In particular, methanol/hydrochloric acid and acetonitrile/trifluoroacetic acid/water are the most employed mixtures; nevertheless, this technique requires extensive time, and the solvent residue is toxic and then represents a limit for the extract’s application, since these are non-GRAS (generally recognized as safe) solvents [45]. Therefore, development of effective methods by using green techniques and GRAS solvents such as ethanol, addressed extraction of polyphenols from cranberries, but even more to conversion of cranberry pomace into higher added value products, is of great importance. In this regard, an alternative method to extract anthocyanins and phenolic compounds from cranberry, and in general thermally sensitive phytochemicals, is the use of subcritical water and pressurized fluids (Table 4). Subcritical water extraction consists of exposing water to high temperatures (above 100 °C), and under enough pressure to remain in the liquid state. Beyond the water, other solvents such as ethanol and mixtures, can be used as pressurized fluids. This is an environmentally friendly technique. At such conditions, the main physicochemical changes include an increase in both ionization and self-diffusivity and a decrease in surface tension [46]. Changes in the temperature and solvent type are critical on the total phenols and anthocyanins, while no significant difference was reported changing pressure. However, the best condition to extract anthocyanins from cranberry is pressurized ethanol at 60–100 °C and 50 bar [27]. In addition to cranberry, this technique has been successfully applied to extract several bioactive compounds, including grape pomace and bilberry [47,48]. Moreover, the choice of acid can influence the stability of anthocyanins and in this contest, organic acids are preferred, and the optimal seems to be hydrochloric acid (2.4 g/100 g cranberries) [46].

There is evidence that many procyanidins are not able to be extracted by conventional methods and they remain in the cranberry pomace, thus, some authors suggest using alkaline hydrolysis to improve the extraction of procyanidins from the treated residues through the breaking of their bonds [27].

A new method proposed by Roopchand and colleagues consists of the extraction of polyphenols from cranberry pomace with 50% (*v*/*v*) aqueous ethanol (at 80 °C, pH 2, for 2 h) and stabilizing the extracted compounds via complexation with soy protein isolate [25]. Nevertheless, it is reported that phenolics can promote the precipitation of the proteins and, consequently, their bioavailability could be reduced. Moreover, a decrease in free phenolic levels may reduce the antioxidant status of the product. In this regard, studies concerning the effect of these interactions on antioxidant capacity showed that a part of the activity is masked, in relation to both the nature of the protein and the phenolics. Therefore, a limitation of the nutraceutical and nutritional value of this type of food cannot be excluded [49].

By co-drying cranberry pomace extract with a protein-rich food matrix, such as soy protein isolate, unlike dried cranberry pomace extract alone, proanthocyanidins, anthocyanins, and total polyphenols were found to be highly stable at 37 °C [25].

The ultrasound-assisted technique is often used in polyphenols extraction [50,51,52] and consists of the shaking of the sample in the extraction solvent for extended periods of time [53]. During the treatment with ultrasounds, the cell wall matrix is disrupted and various compounds, including polyphenols, are released into the medium. The results obtained from ultrasound-assisted extraction showed that this has the greatest potential [45]. Interestingly, Klavins et al. compared the ultrasound, microwave-assisted, and Soxhlet techniques to extract polyphenols from press residues of cranberry. The results confirmed the highest potential of ultrasound-assisted extraction compared with the other methods, being fast, low-cost, and convenient to use. The best extraction solvents were aqueous ethanol and methanol, even if in the presence of acid (trifluoroacetic acid or HCl for anthocyanins or polyphenols respectively), which may limit the use of the final extracts as functional foods or nutraceutical substances [54]. The microwave-assisted technique has also been successfully tested in the extraction of carbohydrates from the pomace of cranberries. Indeed, the total sugars yield reached the value of 21.3% and contained mostly oligosaccharides in the degree of polymerization range of 7 to 10 [55].

Very recently, a supercritical carbon dioxide extraction followed by pressurized fluid extraction has been proposed and tested to obtain a “zero waste” processing of cranberry pomace. Supercritical extraction with carbon dioxide is considered a green technique, since it avoids the use of organic solvents. Moreover, carbon dioxide is a non-toxic, safe, and manageable solvent. It is optimal to extract lipophilic compounds, therefore combined with pressurized fluid extraction, the efficiency of the process can be maximized [56]. 

### 2.4. Clinical Applications

#### 2.4.1. Urinary Tract Infections

UTIs are the second most common type of bacterial infections worldwide, following otitis media [57]. It affects more than 150 million people/year worldwide and causing an economic burden of >$2.6 billion in annual health care expenditures [58].

UTIs are categorized as pyelonephritis and kidney infections when it affects the upper urinary tract (ureters and kidney parenchyma), and as cystitis and urethritis when it affects the lower urinary tract (bladder or urethra). It is also generally divided into sporadic uncomplicated and complicated infections [59]. Complicated UTIs are less common and associated with functional or structural abnormalities such as immunosuppression, urinary obstruction, catheterization, pregnancy, or renal dysfunction [60]. Uncomplicated UTIs are more prevalent in women who have a 50% risk of at least an episode of cystitis (vs. 12% risk in men) during their life and 20–30% risk of recurrent UTIs [61]. Antibiotic therapy represents the most common approach to UTIs, even if it presents some limits including the risk of antibiotic resistance, well documented by the scientific community, and the damage of the intestinal microbiota [62]. In fact, recurrent UTIs require multiple antibiotics for several periods of the year, used also as prophylactic agents. In this regard, the rate of fluoroquinolone resistance is >20% in different nations and the FDA, in 2016, pointed out how the serious side effects associated with fluoroquinolones generally outweigh the benefits for people with uncomplicated UTIs [63]. Relapses in UTIs could be caused by the same microorganism or by a different microorganism. *E. coli* is responsible for 85% of cystitis even if other Gram-negative bacteria such as *Klebsiella pneumoniae* and some Gram-positive bacteria such as *Staphylococcus saprophyticus* and enterococcal species might be implicated in the pathogenesis of uncomplicated UTIs, because it is able to join directly to the bladder epithelium [59,64].

**Figure 2:**
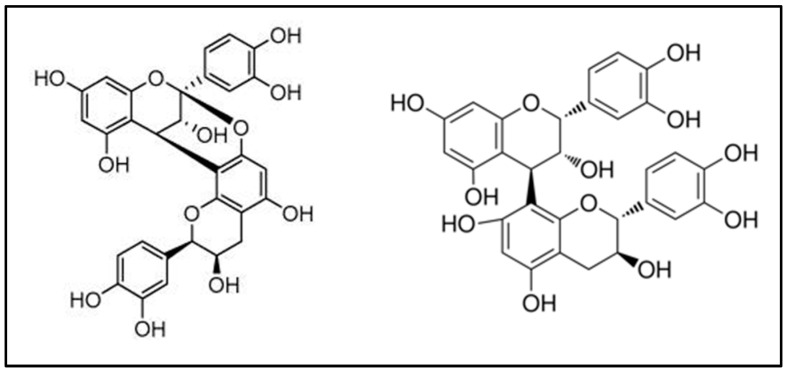
Proanthocyanidin A-type (**left**) and B-type (**right**).

The study by Bodel et al. in 1959 described for the first time the use of cranberry in the prevention of UTIs, attributing its efficacy to the presence of hippuric acid [65]. However, as early as the 1600s, Native Americans used cranberry consumed as a food for the treatment of UTIs and for wound and blood poisoning [66,67]. From the 1990s to 2000s, cranberry research was focused on UTIs prevention, as highlighted by the first robustly designed RCT including 153 females with frequent bacteriuria and randomized to receive 300 mL of cranberry juice or placebo. The results indicated the potential use of cranberry in UTIs, obtaining a reduction by more than 50% of bacteriuria only in the cranberry group [68]. Although the consumption of cranberries has been extensively recommended for UTIs prophylaxis and relief of adverse symptoms, the recommendation coming from the systematic-reviews and meta-analyses of the literature seems to be in part contrasting [69,70,71,72,73,74]. The motive for different conclusions and recommendations of cranberry use in UTIs prevention could be explained by a lack of knowledge regarding the role of cranberry constituents and their impact on urinary tract and gut microbiota, but also the limited characterization of the cranberry materials used in clinical trials, as well as a lack of systematic protocol for the selection of subjects and clinical assays [7]. In fact, cranberry was later found to contain different types of bioactive compounds, including anthocyanins, flavonols and phenolic acids. However, among the polyphenols of this fruit, type-A proanthocyanidins have shown the greatest bioactivity because it is able to inhibit binding of uropathogenic *E. coli* to the uroepithelial cell receptors, attenuate the uropathogen reservoir in the gastrointestinal tract and suppress the inflammatory cascade. In this regard, A-type proanthocyanidins contain an additional ether interflavan bond between C2 →O →C7 if compared with B-type proanthocyanidins, which probably do not exert any effect on UTIs prevention (Figure 2) [75].

**Figure 3:**
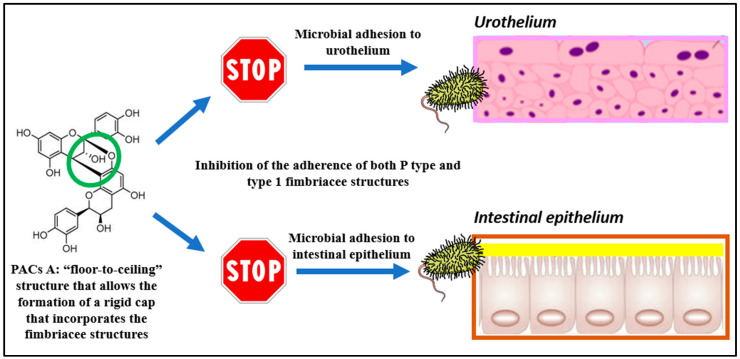
A-type proanthocyanidins (PACs A) and anti-uropathogenic mechanism of action.

A RCT demonstrated the ability of type A proanthocyanidins of cranberry to inhibit the ex vivo adherence of both P-type and type 1 uropathogenic *E. coli* (Figure 3) [76]. A similar result was obtained against *Candida albicans* strain, with a significant reduction in the adherence and biofilm formation after cranberry supplementation [77]. The daily recommended number of PACs to decrease the episodes of UTIs is at least 36 mg [14]. However, cranberry is well known to contain also organic acids (e.g., citric, malic, shikimic, quinic), terpenes, and carbohydrates which might exert pleiotropic activities in UTIs prevention. In particular, D-mannose has been demonstrated to reduce the adherence of *E. Coli* to uroepithelial cells in vitro [78], while vitamin C and other organic acids promote the acidification of the urine with a bacteriostatic effect [79]. Even cranberry xyloglucan oligosaccharides were found to play a role in UTIs prevention, reducing *E. coli* adhesion to the bladder [80].

The meta-analysis by Fu et al., including 7 RCTs conducted on healthy women (*n* = 1498) at risk of UTIs showed that cranberry reduced the risk of UTIs by 26% (pooled risk ratio: 0.74; 95% CI: 0.55, 0.98), suggesting the possible effect of this nutraceutical in preventing uncomplicated UTI recurrence in healthy people [74]. Similar results were obtained by another meta-analysis of 28 RCTs (RR: 0.67, 95% CI 0.55–0.79, *p* < 0.0001) [81]. However, data on complicated UTIs prevention with cranberry remains unclear and in part contrasting as reported by four previous meta-analyses [71,82,83,84], among which only three reported a trend of relative risk reduction after cranberry supplementation [71,82,83].

Cranberry proanthocyanidins and their metabolites also act by reducing the intestine reservoir of potential uropathogenic bacteria. In particular, cranberry flavonoids might decrease the intestinal colonization of opportunistic extra-intestinal *E. coli* and thus, the risk of UTIs incidence [85]. At the same time, the gut microbiota has been shown to interact positively with cranberry flavonoids in a “two-way interaction”, improving the conversion of active metabolites which might enhance the anti-UTI effects and promote the intestinal eubiosis [86]. For example, phenyl-γ-valerolactones are one of the most important metabolites of cranberry found in the urine, with exhibited antiadhesive activity in vitro [87]. Therefore, the heterogeneity of results of cranberry supplementation against UTIs might be also attributed to the inter-variability of gut microbiota composition, which might influence the conversion of cranberry flavonoids into bioactive molecules.

Finally, the combination of cranberry with some probiotic strains (*Lactobacillus* spp.) has been proposed to be effective for the management of recurrent UTIs [88]. *Lactobacillus* spp. seem to improve the production of biosurfactants, bacteriocins, lactic acid, and hydrogen peroxide and to inhibit the intestinal adherence of uropathogenic bacteria [89]. However, RCTs are still inconclusive and need further investigation as a consequence of the small samples size, the different dosages of both cranberry extracts and probiotics, the probiotic strains used, and the typology of enrolled populations [90,91,92].

In conclusion, cranberry supplementation might be used and recommend as adjuvant in order to potentially reduce the incidence of UTIs in particular in people with recurrent infections. Despite evidence suggesting a possible inverse association in the use of cranberry and the need for antibiotics and the economic burden which affects UTIs, data need further confirmation. In addition, both in vitro and long-term clinical trials are necessary to investigate the impact of cranberry supplementation in UTIs relief symptoms, as well as to clarify the mechanisms that contribute to the efficacy of cranberry’s PACs in the reduction of UTIs, the duration of treatments, the dosages of administration, the impact of gut microbiota on the conversion of active metabolites and the differences of the different standardized extracts.

#### 2.4.2. Oral, Gastric and Intestinal Health

Over the last years, several researchers have focused their attention on the effects of cranberry regarding intestinal health for its well-known antiadhesion activity against various microbes in the stomach, small intestine, and colon [93]. However, contrary to the studies about UTIs prevention, the effects of cranberry on the gastrointestinal tract have been mainly investigated with in vitro or animal studies [94].

Cranberry proanthocyanidins act first as anti-inflammatory molecules, reducing the levels of interleukin (IL)-1β, IL-6 [95], bacterial lipopolysaccharide induced expression of iNOS and cyclo-oxygenase-2 (COX-2) in macrophages [96] as well as improving the anti-inflammatory IL-10 [94]. These results were confirmed in a study including mice (eight-week-old) divided into three groups in order to receive a chew diet, a high-fat high-sucrose diet, or high-fat high-sucrose diet, and cranberry extract (0.2 g/Kg) for 8 weeks. At the end of the treatments, cranberry supplementation showed to protect gut inflammation, measured by increasing *Akkermansia* spp. population, reducing the oxidative stress and intestinal triglyceride content [97].

A-type proanthocyanidins and their metabolites also interacted positively with the intestinal microbiota composition, preventing microbial dysbiosis. As demonstrated by Bekiares and colleagues, who investigated the impact of dried cranberries (42 g/day) consumption on human gut microbiota (*n* =10) using the faecal microbiome test, an improvement of *Firmicutes/Bacteroidetes* ratio as well as of the count of *Akkermansia* has been observed after the nutraceutical assumption [98]. In addition, the cranberry powder supplementation (30 g/day) showed to enhance the production of short-chain fatty acids (SCFAs), particularly studied for the post-biotic effect [99].

In the last 15 years, cranberry supplementation was investigated to improve the success of eradication of *H. pylori* infection, which represents the major cause of peptic ulcer disease and gastric cancer [100]. In fact, in vitro studies demonstrated the ability of cranberry constituents to exert anti-adhesion activity on *H. pylori* [101]. In a prospective RCT, 189 Chinese people with *H. pylori* infection were randomly divided to receive cranberry juice (250 mL) or placebo for 90 days. At the end of the study, 14 of the 97 subjects (14.43%) in the cranberry juice treatment group had negative results for the 13C-urea breath test (vs. 5 of the 92 in the placebo group) (*p* < 0.05) [10]. Consumption of high-proanthocyanidins cranberry juice twice daily (44 mg proanthocyanidin/240-mL serving) resulted in decreased *H. pylori* infection rate by 20% as compared with low dosages of proanthocyanidins (*p* < 0.05) [102].

In the study by *Seyyedmajidi and colleagues*, which included 200 patients with *H. pylori* infection and peptic ulcer disease, in treatment with the triple therapy (lansoprazole, clarithromycin, and amoxicillin), the addition of cranberry to triple therapy for *H. pylori* had a higher rate of eradication if compared with the conventional therapy alone (up to 89% and significant) [103]. In another RCT, 177 patients with *H. pylori* infection and treated for the first week with the triple therapy (omeprazole, amoxicillin, and clarithromycin) were randomized to receive 250 mL of either cranberry juice (cranberry + triple therapy, *n* = 89) or placebo (placebo + triple therapy, *n* = 88) twice daily and only cranberry juice or placebo for the next two weeks. At the end of the treatments, analysis by gender revealed that the eradication rate was higher in the cranberry arm for female subjects. However, no significant differences were observed in male subjects [104].

In a multicentric RCT including 295 asymptomatic children (6–16 years of age) who tested positive for *H. pylori*, they were randomly divided into four groups: cranberry juice/Lactobacillus johnsonii La1 (CB/La1), placebo juice/La1 (La1), cranberry juice/heat-killed La1 (CB), and placebo juice/heat-killed La1 (control). Cranberry juice (200 mL) and La1 product (80 mL) were given daily for 3 weeks. At the end of the study, *H. pylori* eradication rates significantly differed in the four groups: 1.5% in the control group compared with 14.9%, 16.9%, and 22.9% in the La1, CB, and CB/La1 groups, respectively (*p* < 0.01) [105]. Finally, an ongoing 4-week study will investigate the effects of cranberry juice fortified with omega-3 intervention on *H. pylori* eradication [106].

Cranberry PACs were demonstrated to prevent the formation of *P. gingivalis* biofilm, and thus to be useful also for oral health. PACs act through three main mechanisms of action: the inhibition of bacterial and host-derived proteolytic enzymes, the regulation of host inflammatory response, and osteoclast differentiation and activity [107]. The studies of *Bodet and colleagues* suggested that cranberry extract has the potential to reduce the proliferation of *P. gingivalis*, *T. forsythia,* and *T. denticola* in periodontal pockets or its protienase mediated destructive processes occurring in periodontitis, reducing the inflammatory cytokine response of macrophages induced by the LPS fraction of Gram- [108,109]. In addition, cranberry has also been shown to reduce the expression of COX-2 and matrix metalloproteinases (MMPs)-1 and -9, produced by resident and inflammatory cells in response to periodontopathogens, such as *Aggregatibacter actinomycetocomitans* [110]. The supplementation of mouthwash cranberry juice in volunteers was demonstrated to reduce the salivary counts of oral streptococci (*S. mutans*), acting as an oral anti-adhesive nutraceutical and inhibiting the extracellular polysaccaride synthesis that promotes the sucrose dependent adhesion of oral bacteria [111,112]. Similar results were shown by *Yamanaka* et al. [113], *Durate* et al. [114], and *Weiss* et al. [115] who examined the effects of cranberry polyphenols (supplemented through mouthwashes, tooth paste, or chewing gum) on biofilm formation and bacterial growth of *S. mutans*. The research groups concluded by highlighting the possible preventive action of cranberry polyphenols in the development of dental plaque.

The impact of cranberry supplementation on oral health, gut microbiota, and *H. pylori* eradication represents one of the most interesting research points of the coming years, in order to prevent oral health and both gastro-intestinal and extra-intestinal diseases. However, longer RCTs are still lacking and urgent to better understand the role of bioactive molecules of cranberry in oral and gut microbiome modulation and the future perspectives for this nutraceutical beyond the UTIs prevention.

#### 2.4.3. Cardiometabolic Effects

Cardiovascular diseases (CVDs) are the leading cause of mortality and disability in developed countries [116]. The impact of CVDs is estimated to have been US $906 billion in 2015 and is expected to rise by 22% by 2030 [117]. In this sense, cranberries which are a rich source of proanthocyanidins, anthocyanins, flavanols, flavonols, and phenolic acids, may contribute to reducing or preventing some cardiovascular modifiable risk factors [118]. However, to date, despite several studies have explored the effect of cranberry supplementation on CV risk factors in different categories of people (healthy subjects [119], patients with type 2 diabetes [120], with coronary artery disease [121], overweight or metabolic syndrome [122]), the produced results have been contrasting and in part remain unclear.

24 Chinese people with type 2 diabetes and treated with 160 mg/day of anthocyanins from bilberries and blackcurrants showed a reduction in fasting plasma glucose and HOMA-IR [123]. Similar results were obtained with cranberry juice (240 mL/day) consumed by overweight, older adults [119]. In another randomized and placebo-controlled pilot study, 8 overweight or obese men and women with abdominal adiposity consumed 450 mL/day high polyphenol cranberry extract beverage or placebo for 8 weeks. The results showed a reduction of endothelin-1, fasting C-reactive protein and reduced oxidized glutathione ratio, and an improvement of nitric oxide (*p* < 0.05 for all) compared with the placebo. Cranberry also reduced serum insulin and increased HDL cholesterol compared with the placebo (*p* < 0.05) [118].

However, data on insulin improvement after cranberry treatments are still contrasting, as highlighted in a study that investigated the effects of cranberry juice in subjects with coronary artery disease (no improvement of fasting glucose, insulin, or HOMA-IR) [121]. In a RCT, 35 obese individuals with elevated fasting glucose or impaired glucose tolerance were treated with 450 mL/day of low-calorie cranberry beverage or placebo for 8 weeks. At the end of the study, a significant reduction of lipid peroxidation (measured as levels of 8-isoprostane group) was shown in the cranberry group (−2.18 pg/mL), while it was increased in the placebo group (+20.81 pg/mL) (*p* = 0.02). In addition, an improvement in levels of triglycerides (−13.75% vs. +10.32%; *p* = 0.04) and nitrates (+3.26 µM/L vs. −6.28 µM/L; *p* = 0.02) was also observed. However, cranberry beverage consumption showed no impact on insulin sensitivity [124]. Another RCT, partially in contrast with this finding, included 41 overweight patients with non-alcoholic fatty liver disease who were randomly allocated to receive either a cranberry supplement (288 mg/day of extract, equivalent to 26 g of dried cranberry) or a placebo for 12 weeks. At the end of the study, alanine aminotransferase and insulin decreased significantly especially in the active group (alanine aminotransferase: *p* < 0.05 compared with placebo; insulin: *p* < 0.001 compared with baseline for active group and *p* = 0.020 compared with baseline with placebo) [125].

Cranberry supplementation has no lipid-lowering properties as demonstrated by several RCTs, even if anthocyanins have been reported to improve LDL cholesterol in dyslipidemic people through the inhibition of cholesterol ester transfer protein [118]. Data on HDL cholesterol improvement remains still contrasting. Ruel et al. reported an improvement by 8% of HDL cholesterol after cranberry juice consumption in people with abdominal adiposity [126]. Other studies reported no significant effects on lipid profile [121,122,127,128]. Nevertheless, differences in the duration of the studies, populations, baseline subject characteristics, medication use, polyphenol composition of the juice may underline the great heterogeneity of the results obtained. An interesting ability of cranberry, due to its antioxidant capacity, is the reduction of biomarkers of oxidative stress. Cranberry juice supplemented in people with metabolic syndrome [12] as well as in healthy subjects [128] demonstrated to reduce oxidized LDL (which contributes to the progression of atherosclerosis), inflammatory markers (hs-CRP, endhotelin-1) [119], and increase the nitric oxide (NO) release suggesting a temporal benefit to vasodilation [129,130].

In conclusion, to date, data regarding the supplementation of cranberry on glycemia and insulinemia is still contrasting and needs further investigations also to understand the glucoregolation mechanism of actions. Cranberry seems not able to exert significant lipid-lowering activities even if it may contribute to reducing lipid peroxidation, oxidative stress, and inflammation.

## 3. Discussion and Future Perspectives

Today, the first indication for cranberry prescription regards the prevention of UTIs, which are responsible for 7 million doctor visits, 1 million emergency room visits, and 100,000 hospitalizations each year (only in the USA), with an estimated economic burden of $1.6 billion [131]. In this context, based on the results of evidence present in the literature, cranberry supplementation could be used and recommend in order to reduce the incidence of UTIs in particular in people with recurrent infections, and reducing the use of antibiotics in people with UTIs. However, the impact of cranberry supplementation in UTIs relief symptoms is still unclear and in part contrasting. Even the mechanisms which contribute to the efficacy of cranberries’ PACs in UTIs prevention and the impact of gut microbiota on the conversion of active metabolites need to be clarified. Possible cranberry indications beyond UTIs include the prevention of oral health, intestinal eubiosis, and the *H. pylori* eradication in addition to the conventional treatments. In this regard, despite that it represents one of the most interesting research points of the coming years, longer RCTs are urgent to better understand the role of bioactive molecules of cranberry in oral and gut microbiome modulation before recommending it in clinical practice [102]. Similar discourse concerns the use of cranberry in cardiovascular prevention where it seems to contribute to reducing lipid peroxidation, oxidative stress, and inflammation [119].

An important limitation that concerns cranberry supplementation is the generally high dropout rates which can exceed 30% especially for sugar-free juices (very-low palatable) [132]. In this regard, new investigations and formulative strategies are important to improve compliance and persistence to treatment.

Another important limit in cranberry supplementation regards the high costs to obtain titrated (in PACs) and standardized extracts. The above-mentioned conventional techniques do not allow the production of inexpensive quality extracts and thus low-cost strategies to prevent UTIs and other diseases [133]. In the cost-effectiveness analysis by van den Hout and colleagues, including high-UTI-risk residents, the supplementation of cranberry capsules was demonstrated to be effective in preventing UTIs but was not likely to be cost-effective in the investigated dosage, frequency, and setting [134]. In this regard, not all cranberry products have been shown to comply with the laws of European and non-European countries. The study conducted by *Wang* et al. and *Mannino* et al. emphasized the urgency of standardized product quality control and labelling for cranberry dietary supplements manufacture and marketing, reporting some cases of adulteration by other botanical extracts, non-uniformity between the test of dosage forms and the contents of PACs, and the low quality of extracts [135,136].

The problem of cheap but poor-quality cranberry extracts is a serious, underestimated, and potentially dangerous problem for consumer health, not only for the absence of PACs or for the low-quality un-titrated extracts, but above all for the presence of contaminants. A Chinese study that used the gas chromatography-triple quadrupole tandem mass spectrometry demonstrated a possible contamination with pesticide residues of cranberry plant extracts [137]. However, the EFSA Panel report based on cranberry consumption history, clinical trials, description of manufacturing processes as well as the extracts composition concluded that this product is safe as a nutraceutical and as a food ingredient. Safety data including long-term studies is still lacking and needs further investigations [138].

An important challenge for the next years is also to develop a food-compatible method for the extraction of cranberry phytochemicals and thus produce qualitatively effective and safe extracts. The study by *Klavins* et al. demonstrated the great potential of unconventional techniques such as ultrasound or microwave-assisted extraction associated with hydroalcoholic solvents, being potentially fast to use and ensuring good yields [54]. Nevertheless, despite the rich literature on non-conventional techniques to recover bioactive compounds such as the PACs, the industrial-scale production remains an open challenge. In this context, the continuous availability of cranberries and the isolation and extraction of selective bioactive components represent still a barrier to scale-up.

The ideal extraction technique for cranberry PACs should enable a low energy consumption using water as a solvent, resulting in high yields, little capital investment, and easy integration into current processing lines. However, none of the unconventional techniques described in the literature really satisfy all of these points. In fact, although the unconventional techniques, such as ultrasound or microwave-assisted extraction have been shown to improve the extraction efficiency, their cost remains high if compared to classical methods, and new solutions to reduce these constraints are, at this moment, still lacking or unpracticable.

## 4. Conclusions

Cranberry extracts supplementation as a functional food and nutraceutical, could constitute an adjuvant for the prevention of urinary tract infections, and probably may exert benefits in the prevention of cardiovascular and gastroenteric diseases. Aiming to validate the efficacy and safety of clinical applications as long-term RCTs, further investigations of the mechanisms of action are required. The quality of cranberry’s polyphenolic fractions start from the raw material, then extraction processing and formulation and represents a key point for a good food supplement. New non-conventional extraction methods are welcome to improve the quality of the extracts and reduce the overall costs.

## Figures and Tables

**Table 1:** Polyphenols content of cranberry foods (adapted from Blumberg et al. 2013 [14]).

Food Source		Flavan-3-ol Monomers and Dimers [11,15]	Proanthocyanidins [11,16]	Anthocyanins [17,16]	Hydroxybenzoic Acids [15,18]	Hydroxycinnamic Acids [15,18]	Terpenes [19]	Flavonols [18]
Cranberry fruit	mg/100 g	7–33	133–367	13–171	503–602	73–82	65–125	20–40
	mg/serving (80 g whole fruit)	5.6–26.4	106–293	10.4–136.8	402–482	57.6–65.6	52–100	16–32
Cranberry juice	mg/100 g	6–35	89–230	27–132	64	12–19	Trace	11–58
	mg/serving (200 mL juice)	7	17.8–46	5.4–26.4	12.8	2.4–3.8	Trace	2.2–11.6
Canned cranberry sauce	mg/100 g	112.8	16–54.4	0.6–11.8	476	47.5	1.1–22.8	—
	mg/serving (70 g sauce)	78.9	11.2–38	0.4–8.3	333.2	33.2	0.8–16	—
Sweetened, dried cranberries	mg/100 g	—	64.2	10.3	—	—	98.5	—
	mg/serving (40 g dried fruit)	—	25.6	4.1	—	—	39.4	—

**Table 2:** Content of polyphenolic compounds in cranberry pomace (adapted from White et al. 2011 [27]).

Polyphenols	Concentration (mg/100 g dw)
Anthocyanins	
Cyanidin 3-arabinoside	49.6 ± 6.8
Peonidin 3-arabinoside	26.6 ± 0.5
Peonidin 3-galactoside	20.1 ± 0.5
Cyanidin 3-galactoside	13.2 ± 0.2
Peonidin 3-glucoside	7.4 ± 0.3
Cyanidin 3-glucoside	4.5 ± 0.2
Total anthocyanins	121.4 ± 5.9
Flavonols	
Quercetin	146.2 ± 22.7
Myricetin	55.6 ± 2.6
Quercetin 3-benzoyl galactoside	27.5 ± 3.4
Quercetin 3-rhamnoside	18.5 ± 3.4
Quercetin 3-arabinofuranoside	16.7 ± 3.5
Quercetin 3-arabinopyranoside	15.2 ± 3.6
Quercetin 3-galactoside	12.8 ± 3.6
Unidentified	12.1 ± 3.5
Methoxyquercetin 3-xyloside	11.4 ± 3.7
Quercetin 3-xyloside	5.5 ± 0.3
Quercetin 3-coumaroyl galactoside	2.3 ± 0.3
Myricetin 3-arabinoside	1.8 ± 0.1
Myricetin 3-xyloside	1.5 ± 0.3
Total flavonols	358.4 ± 16.3
Procyanidins	
Dimer (DP2)	52.7 ± 1.7
Trimer (DP3)	30.7 ± 1.4
Hexamer (DP6)	25.6 ± 1.2
Pentamer (DP5)	22.7 ± 1.2
Heptamer (DP7)	16.6 ± 1.2
Octamer (DP8)	16.1 ± 2.9
Tetramer (DP4)	16.1 ± 1.3
Nonomer (DP9)	13.2 ± 1.1
Monomer (DP1)	5.12 ± 0.0
Total procyanidins	186.5 ± 8.8

**Table 3:** Content of polyphenolic compounds depending on cranberry processing step and type of pre-treatment (adapted from White et al. 2011 [27]).

Processing Step	Pre-Treatment	Polymer Concentration (mg/100 g Fresh Berries)	Polymer %
Fresh	-	206.2 ± 9.3	81.7
Blanched	Grinding + blanching	251.8 ± 8.4	80.7
	No grinding + blanching	245.3 ± 18.5	82.6
Enzyme treated mash	Grinding + blanching	261.4 ± 20.9	81.2
	Grinding + no blanching	319.6 ± 11.5	85.3
	No grinding + blanching	172.7 ± 8.6	76.1
Unclarified juice	Grinding + blanching	104.2 ± 17.7	82.3
	Grinding + no blanching	103.5 ± 8.2	76.0
	No grinding + blanching	100.7 ± 4.3	78.5
Clarified juice	Grinding + blanching	107.7 ± 3.6	80.4
	Grinding + no blanching	74.0 ± 10.9	67.5
	No grinding + blanching	86.2 ± 4.5	78.0
Pasteurized juice	Grinding + blanching	76.1 ± 4.0	76.5
	Grinding + no blanching	69.4 ± 2.7	68.0
	No grinding + blanching	75.3 ± 7.2	73.6
Pomace	Grinding + blanching	109.7 ± 1.7	88.9
	Grinding + no blanching	127.7 ± 3.9	92.5
	No grinding + blanching	130.4 ± 0.5	90.0

**Table 4:** Advantages and limits of polyphenols extraction from cranberry using classic or non-conventional techniques.

	Technique	Advantages	Limits	References
Conventional methods	Juice	Low costs	Food industry waste (pomace)	[24,29,30]
	Pressing or decanting from frozen berries	Low costs		
	Drying process	Low costs	Exposition of cranberry fruits to high temperature and air for long time. Partial loss of nutritional value	
	Spray-drying evaporation	Enriched amount of poliphenols	Variable concentration of polyphenols, depending on enzymes, temperature and time conditions and the concentration and drying steps	[7]
Non-conventional methods	Subcritical water extraction	Selectivity and purity of the final extract. Environmentally friendly Efficacy of extraction of other biactive molecules. Fast and low-cost.	High energy for water evaporation	[46]
	Ultrasound-assisted extraction	Short extraction time	Scalability mainly in flow mode	[37,45]
	Microwave-assisted extraction	Selectivity and purity of the final extract.	Limited to MW-adsorbing mixtures	[37,55]
	Supercritical carbon dioxide extraction followed by pressurized fluid extraction	Environmentally friendly, easy scalability	High CAPEX	[56]

## Data Availability

Not applicable.
